# No-preparation and conventional veneers in esthetic dentistry: Clinical considerations

**DOI:** 10.6026/973206300221560

**Published:** 2026-03-31

**Authors:** Amisha Chandode, Amit Hemraj Patil, Arshia Rashid Baig, Himmat Jaiswal, Sheetal Mali, Isha Antony

**Affiliations:** 1 Department of Conservative Dentistry and Endodontics, Bharati Vidyapeeth Dental College and Hospital, Bharati Vidyapeeth (Deemed to be) University, Navi Mumbai, Maharashtra, India; 2 Department of Conservative Dentistry and Endodontics, Sharad Pawar Dental College and Hospital, Sawangi (Wardha), Maharashtra, India

**Keywords:** Ceramics, dental veneers, esthetics, dental, minimally invasive dentistry, porcelain, tooth preparation techniques

## Abstract

The choice between no-preparation and conventional porcelain veneers remains a clinical challenge due to the differences in clinical longevity, esthetic outcomes and preservation of tooth structure. Therefore, it is of interest to compare no-preparation porcelain veneers with conventional porcelain veneers to support clinical decision-making. Evidence from peer-reviewed studies demonstrates favorable short- to mid-term survival of no-preparation veneers. Success rates reach up to 97.4% when bonded to enamel in well-selected cases. The advantages of no-preparation veneers include enamel preservation, reduced postoperative sensitivity and have a low biological cost. However, they are not recommended for cases with intrinsic discoloration, misalignment, or significant morphological discrepancies. Conventional veneers remain dependable for complex esthetic corrections; however, they require greater tooth reduction and carry a higher biological risk.

## Background:

Advances in adhesive dentistry and ceramic materials have made porcelain laminate veneers a reliable choice for anterior tooth esthetic rehabilitation [1]. Conventional veneer techniques usually require precise enamel reduction. In some cases, dentin reduction may be required to accommodate ceramic thickness and achieve adequate retention [2]. Long-term studies have shown favorable survival rates for conventional veneers [3]. However, these procedures result in irreversible loss of tooth structure and may cause postoperative sensitivity when dentin is exposed. To overcome these limitations, no-preparation and minimally invasive veneer techniques have been developed, showing a growing emphasis on preserving enamel and using biologically conservative methods [4]. These ultra-thin restorations primarily bond to sound enamel, which improves adhesive reliability and may lower the need for anesthesia and temporary restorations [5]. The traditional clinical practices for no-preparation veneers highlight the need for proper case selection, precise margin control and appropriate management of the emergence profile to avoid over-contouring [6]. However, despite their advantages, the long-term clinical performance of no-preparation and minimally invasive veneers over time remains less clearly understood compared to that of conventional veneers [7]. The variability in reported survival and success rates is mainly due to differences in study design, outcome criteria and follow-up duration [8]. Therefore, it is of interest to report current clinical evidence on the indications, limitations and clinical performance of no-preparation and conventional porcelain veneers.

## Indications for minimal preparation and conventional veneers:

Due to the growing emphasis on enamel preservation, the discussion about minimal preparation veneers (MPVs) versus conventional veneers (CVs) remains an important topic in restorative dentistry [1]. Compared to MPVs, CVs remain the first choice for treating the most difficult discolorations, morphological alterations and alignment deviations since controlled tooth reduction enhances the ability to mask the tooth and the accuracy of the contour [2, 3]. Hence, in such cases, it is crucial to have the right plan of tooth preparation in order to get a predictable esthetic result. MPVs, acknowledging their minimal intervention approach, have been promoted for cases with good alignment and minimal esthetic requirements. Long-term clinical trials show good survival results when bonded only to enamel. This strongly suggests clinical success depends heavily on an appropriate case selection [4].

## Role of enamel bonding and preparation design:

These research results strongly support the critical role that sound enamel has in obtaining a dependable adhesive bond and a durable restoration over time. Preparation design is the most important deciding factor of the clinical outcomes for MPVs [1]. This usually follows an additive approach with little to no enamel removal. This preserves natural tooth contours and minimizes the need for anesthesia or provisional restorations [3].

## Influence of preparation margins on longevity:

Conventional veneers require more tooth reduction. They have been proven to be long lasting when the preparations are only to enamel and the adhesive protocols are well followed [6]. No-preparation veneers preserve enamel without reduction and suit mild esthetic cases, whereas conventional veneers require 0.3-1.0 mm reduction for improved masking and contour control [9]. The extension and the location of the preparation margins are important factors determining the longevity of the restoration.

## Variability in survival rates:

It is quite difficult to compare survival rates of veneers between studies due to differences in study design and outcome definitions [7]. A recent systematic review noted that variation in study design and outcome measures contributes to the wide range of survival rates reported for porcelain laminate veneers [10]. Some studies use stringent survival criteria. Even trauma-related events are considered failures. This lowers reported survival rates and limits comparisons [4]. When evaluating data on survival, this methodological variability must be considered. Both techniques show high survival (91-100%), with no-preparation veneers demonstrating slightly higher mean survival (97% vs 92%) and fewer catastrophic failures [9].

## Esthetic outcomes and clinical limitations:

From an esthetic perspective, minimal preparation veneers (MPVs) are very well accepted by patients. They involve less treatment discomfort and better optical integration provided that the patient is properly selected [8]. Yet MPVs are not the answer to every case and may lead to less esthetic results if heavy color masking or contour change is needed. In such scenarios, conventional veneers (CVs) would still be the treatment of choice as they offer wider restorative options and a more predictable esthetic correction [3].

## Complementary role of veneer techniques:

The existing data reveal that the application of enamel-based, minimally invasive veneer techniques can be enhanced while the use of conventional preparations in complicated clinical situations is still warranted [1,6]. Hence, MPVs and CVs should not be considered as opposing techniques but rather as complementary approaches depending on patient-specific indications [5]. A summary of representative studies comparing clinical longevity, esthetic outcomes and tooth preservation is presented in Table 1.

## Conclusion:

Both minimal preparation veneers (MPVs) and conventional veneers show good clinical and esthetic results when appropriately indicated. MPVs offer advantages in enamel preservation, adhesive bonding and patient acceptance in carefully selected cases, while conventional veneers remain essential for complex esthetic cases requiring significant morphological or discoloration correction. Current evidence supports a patient-specific, enamel-centered treatment approach that combines minimally invasive principles with the continued relevance of traditional preparation methods.

## Advancement to knowledge:

This review consolidates current clinical evidence comparing no-preparation and conventional porcelain veneers with respect to clinical longevity, esthetic outcomes and tooth structure preservation. It provides clarity on their indications and limitations to support informed treatment planning.

## Figures and Tables

**Table 1:** Comparison of clinical longevity, esthetic outcomes and tooth preservation between veneer techniques

**Author (Year)**	**Study type**	**Clinical longevity**	**Esthetic outcomes**	**Tooth Preservation**
Ali (2023) [1]	Systematic review	Higher survival reported for enamel-bonded veneers	Esthetic outcomes influenced by preparation depth and bonding substrate	Enamel preservation associated with improved adhesive reliability
Smielak *et al.* (2022) [2]	Prospective clinical study (9 years, 186 veneers)	MPVs: 100% survival; CVs: 9.67% survival under strict Kaplan-Meier criteria	Stable surface texture with appropriate finishing	MPVs limited to enamel (0-0.3 mm); CVs frequently extend into dentin
Aslan *et al.* (2019) [3]	Long-term clinical case series (10-20 years, 413 veneers)	CVs: 95% (10 years), 91% (15 years), 87% (20 years)	Long-term esthetic stability with standardized adhesive protocols	Longevity dependent on conservative preparation design
Gurel *et al.* (2013) [4]	Retrospective study (12 years, 580 veneers)	CVs: 86% overall survival; 99% survival with enamel margins	CVs suitable for complex esthetic correction	Dentin margins associated with increased failure risk
De Angelis *et al.*(2021) [5]	Retrospective clinical evaluation (2.5-5 years, 76 restorations)	MPVs: 97.4% survival; failures mainly in high-load canines	94% margins rated excellent or good; minimal marginal discoloration	Enamel preserved; postoperative sensitivity uncommon
D'Arcangelo *et al.* (2018) [6]	Clinical protocol for no-preparation veneers	Not assessed	Natural emergence profile achieved with appropriate case selection	Biologically conservative approach with minimal sensitivity
Tuncdemir *et al.* (2020) [8]	*In-vitro* evaluation (color stability)	Not assessed	MPVs show minimal color change; CVs exhibit greater discoloration after aging	Preparation depth influenced esthetic aging
Note: Table compiled by the authors as a
summary of published literature; no table has been
reproduced from original sources
Note: Survival rates across studies are not directly
comparable due to differences in study design and outcome definitions.
